# Characterization of biventricular alterations in myocardial (reverse) remodelling in aortic banding-induced chronic pressure overload

**DOI:** 10.1038/s41598-019-39581-9

**Published:** 2019-02-27

**Authors:** Daniela Miranda-Silva, Patrícia Gonçalves-Rodrigues, João Almeida-Coelho, Nazha Hamdani, Tânia Lima, Glória Conceição, Cláudia Sousa-Mendes, Cláudia-Moura, Arantxa González, Javier Díez, Wolfgang A. Linke, Adelino Leite-Moreira, Inês Falcão-Pires

**Affiliations:** 1 Department of Surgery and Physiology, University of Porto, Porto, Portugal; 2 Department of Systems Physiology, Ruhr University, Bochum, Germany; 3 Institute of Physiology II, University of Muenster, Muenster, Germany; 4 Program of Cardiovascular Diseases, Centre for Applied Medical Research, University of Navarra and CIBERCV, Pamplona, Spain; 5 Department of Cardiology and Cardiac Surgery and Department of Nephrology, University of Navarra Clinic, Pamplona, Spain

## Abstract

Aortic Stenosis (AS) is the most frequent valvulopathy in the western world. Traditionally aortic valve replacement (AVR) has been recommended immediately after the onset of heart failure (HF) symptoms. However, recent evidence suggests that AVR outcome can be improved if performed earlier. After AVR, the process of left ventricle (LV) reverse remodelling (RR) is variable and frequently incomplete. In this study, we aimed at detecting mechanism underlying the process of LV RR regarding myocardial structural, functional and molecular changes before the onset of HF symptoms. Wistar-Han rats were subjected to 7-weeks of ascending aortic-banding followed by a 2-week period of debanding to resemble AS-induced LV remodelling and the early events of AVR-induced RR, respectively. This resulted in 3 groups: Sham (n = 10), Banding (Ba, n = 15) and Debanding (Deb, n = 10). Concentric hypertrophy and diastolic dysfunction (DD) were patent in the Ba group. Aortic-debanding induced RR, which promoted LV functional recovery, while cardiac structure did not normalise. Cardiac parameters of RV dysfunction, assessed by echocardiography and at the cardiomyocyte level prevailed altered after debanding. After debanding, these alterations were accompanied by persistent changes in pathways associated to myocardial hypertrophy, fibrosis and LV inflammation. Aortic banding induced pulmonary arterial wall thickness to increase and correlates negatively with effort intolerance and positively with E/e′ and left atrial area. We described dysregulated pathways in LV and RV remodelling and RR after AVR. Importantly we showed important RV-side effects of aortic constriction, highlighting the impact that LV-reverse remodelling has on both ventricles.

## Introduction

Ventricular remodelling includes structural and functional changes taking place in the ventricle in response to chronic pressure overload. Aortic stenosis (AS) is the most common valvulopathy, whereby a stenotic valve increases afterload and imposes additional hemodynamic stress on the left ventricle (LV). LV overload activates several molecular and cellular pathways that trigger remodelling through morphological and functional alterations^1,2^. One of these changes is ventricular hypertrophy, which is perceived as an initial compensatory mechanism to normalise increased wall stress. However, over time LV hypertrophy becomes decompensated^3^ and results in diastolic dysfunction (DD), with an increase in LV stiffness, an abnormal filling and relaxation pattern, followed by an enlargement of the left atrium and pulmonary congestion^4^. Myocardial maladaptive remodelling is one of the principal pathological markers of cardiovascular disease progression/severity, and its prevention or reversal is a desirable strategy. Currently, the most effective treatment to AS is the surgical alleviation of pressure overload subsequently to aortic valve replacement (AVR). AVR allows the myocardium to undergo a process named reverse remodelling (RR), which usually results in an improvement of cardiac structure and function. However, the process of RR is frequently incomplete, and the underlying mechanisms remain to be clarified as patients show an extremely variable myocardial response during RR, ranging from partial to total recovery of cardiac function and structure.

Incomplete functional recovery is an indicator of poor prognosis, associated with persisting symptoms and increased mortality^5^. Diastolic dysfunction, associated with impaired active relaxation, is a feature of most AS patients. Several studies showed improvement of diastolic function^6,7^ including active relaxation early after AVR^8^, however, the percentage of patients with moderate to severe DD increases 10-years after AVR^9^.

In incomplete structural recovery after AVR, AS patients with greater LV mass regression associate positively with lower rates of rehospitalisation^10^. Indeed, LV mass regression, which usually does not exceed 31% at 6 months post-AVR, is assumed to be a favourable marker of LV RR^11,12^.

RV failure is a frequent complication following LV assist device (LVAD) implantation^13^, and in AS deterioration of RV function after AVR has been shown^14^. Nevertheless, knowledge about RV function and structure in this context remains scarce, especially in a modulable experimental context.

Currently, most of the cellular and molecular information regarding RR derives from samples of patients undergoing RR after LVAD implantation in which myocardial sample are available before and after unloading the heart. However, these “end-line” patients suffer from advanced heart failure (HF) characterised by chamber dilatation and reduced ejection fraction (EF), and therefore they do not represent the typical phenotype of AS-induced myocardial remodelling. Thus, we aimed to describe the molecular pathways underlying myocardial remodelling in the presence of DD and preserved EF as well as to highlight the early biventricular changes invoked by myocardial RR focusing on cardiomyocytes’ myofilaments, calcium-handling, signalling pathways and extracellular matrix. To achieve these goals, we selected a rat model of aortic banding, which is widely used to impose chronic pressure overload, therefore mimicking AS-induced remodelling during increased afterload, followed by aortic debandingto trigger RR upon overload relief^15,16^.

## Results

### Left ventricle cardiac structure and functional characterisation

Echocardiography of banded animals revealed ventricular concentric hypertrophy, based on thicker LV wall and decreased LV dimensions (Table 1). In response to LV overload, hypertrophy was associated with increased systolic and diastolic LV pressures as well as with augmented arterial elastance. Diastolic dysfunction was evident by the significant decrease of E/A, increased E/E´, LVEDP, Tau, EDPVR and left atrial dilatation. Regarding systolic function, Ba animals presented preserved EF and increased contractility (ESPVR, Table 1).

**Table 1:** Left Ventricle morphological and functional data.

	Sh (n = 10)	Ba (n = 15)	Deb (n = 10)
**Echocardiography Evaluation**			
BSA (cm^2^)	4.873 ± 0.035	4.947 ± 0.080	4.916 ± 0.080
AWd (cm)	0.143 ± 0.006	0.202 ± 0.005^αααα^	0.168 ± 0.010^χχ^
LVd (cm)	0.743 ± 0.013	0.696 ± 0.014^α^	0.769 ± 0.016^χχ^
PWd (cm)	0.138 ± 0.007	0.206 ± 0.008^αααα^	0.166 ± 0.008^αχχ^
AWs (cm)	0.220 ± 0.005	0.306 ± 0.008^αααα^	0.252 ± 0.012^αχχ^
LVs (cm)	0.448 ± 0.025	0.383 ± 0.013^α^	0.467 ± 0.029^χχ^
PWs (cm)	0.216 ± 0.005	0.307 ± 0.011^αααα^	0.263 ± 0.010 ^ααχ^
LVMass (g)	0.688 ± 0.035	1.070 ± 0.032^αααα^	0.908 ± 0.037^ααχχ^
EDVI (µL.cm^−2^)	187.119 ± 8.217	165.833 ± 8.434^α^	205.797 ± 13.445^χχ^
ESVI (µL.cm^−2^)	42.437 ± 4.727	29.874 ± 2.797^α^	50.469 ± 8.388^χχ^
E/A	1.547 ± 0.074	1.354 ± 0.041^α^	1.571 ± 0.107^χ^
E/E′	13.456 ± 0.694	18.635 ± 0.995^αα^	14.667 ± 0.702^χ^
EF (%)	74 ± 4	78 ± 3	74 ± 3
LAA (cm^2^)	0.314 ± 0.015	0.421 ± 0.021^ααα^	0.370 ± 0.011^α^
Ao. Velocity (m.s^−1^)	1.53 ± 0.19	4.14 ± 0.21^αααα^	2.3 ± 0.15^αχχχχ^
**Haemodynamic data**			
LVSP (mmHg)	113.47 ± 3.39	187.64 ± 11.62^αααα^	123.7 ± 3.82^χχχ^
LVEDP (mmHg)	4.68 ± 1.13	10.63 ± 1.70^αα^	5.11 ± 0.79^χχ^
HR (bpm)	381 ± 15	399 ± 8	410 ± 12
EA (mmHg.µL^−1^)	0.70 ± 0.08	1.07 ± 0.14^αααα^	0.90 ± 0.12^ααχχ^
ESPVR (mmHg.µL^−1^)	0.47 ± 0.16	1.16 ± 0.35^αα^	0.44 ± 0.09^χ^
EDPVR (mmHg.µL^−1^)	0.014 ± 0.004	0.031 ± 0.008^α^	0.023 ± 0.05
Tau (ms)	7.57 ± 0.42	9.18 ± 0.55^α^	7.60 ± 0.34^χ^

Body surface area; **AWd**, anterior wall in diastole; **LVd**, left ventricle cavity in diastole; **PWd**, posterior wall in diastole**; AWs**, anterior wall in systole; **LVs**, left ventricle cavity in systole; **PWs**, posterior wall in systole; **LVMass**, left ventricle mass; **EDVI**, end-diastolic volume index; **ESVI**, end- systolic volume index; **HR**, heart rate; **E′**, wave velocity of tissue Doppler at the lateral mitral annulus; **E**, peak of pulse Doppler wave of early mitral flow velocity**; A**, peak of pulse Doppler wave of late mitral flow velocity**; E/A**, ratio between peak E and A waves; **E/E′** ratio between E and E′ waves; **TEI index**, myocardial performance index; **EF**, ejection fraction; **LAA**, left atrium area; **Ao**. **Velocity**, **aortic velocities**; **LVSP**, left ventricular systolic pressure; **LVEDP**, left ventricle end-diastolic pressure; **HR**, heart rate; **ESPVR**, end-systolic pressure volume relationship; **EDPVR**, end-diastolic pressure-volume relationship; **Tau**, constant of relaxation. Values are mean ± SEM. One Way-ANOVA, **Ba/Deb vs Sh:** ^α^p < 0.05; ^αα^p < 0.01; ^ααα^p < 0.001; **Deb vs Ba**: ^χ^p < 0.05; ^χχ^p < 0.01, ^χχχ^p < 0.001.

The debanding group showed an overall imrpovement in haemodynamic parameters (LVSP, LVEDP, ESPVR, EDPVR and Tau) and partial regression of hypertrophy as confirmed by thinner LV wall and decreased LV mass compared to banding rats. Some of these variables did not normalise completely, persisting significantly different from the sham group (Table 1). In the debanding rats, EDPVR decreased to intermediate values between banding and sham group. Aortic velocity was attenuated after aortic constriction relief andcorrelates with some LV and RV systolic and diastolic parameters (Fig. S2).

### Left ventricle cardiomyocytes hypertrophy, passive tension and extracellular matrix remodelling

**Figure 1:**
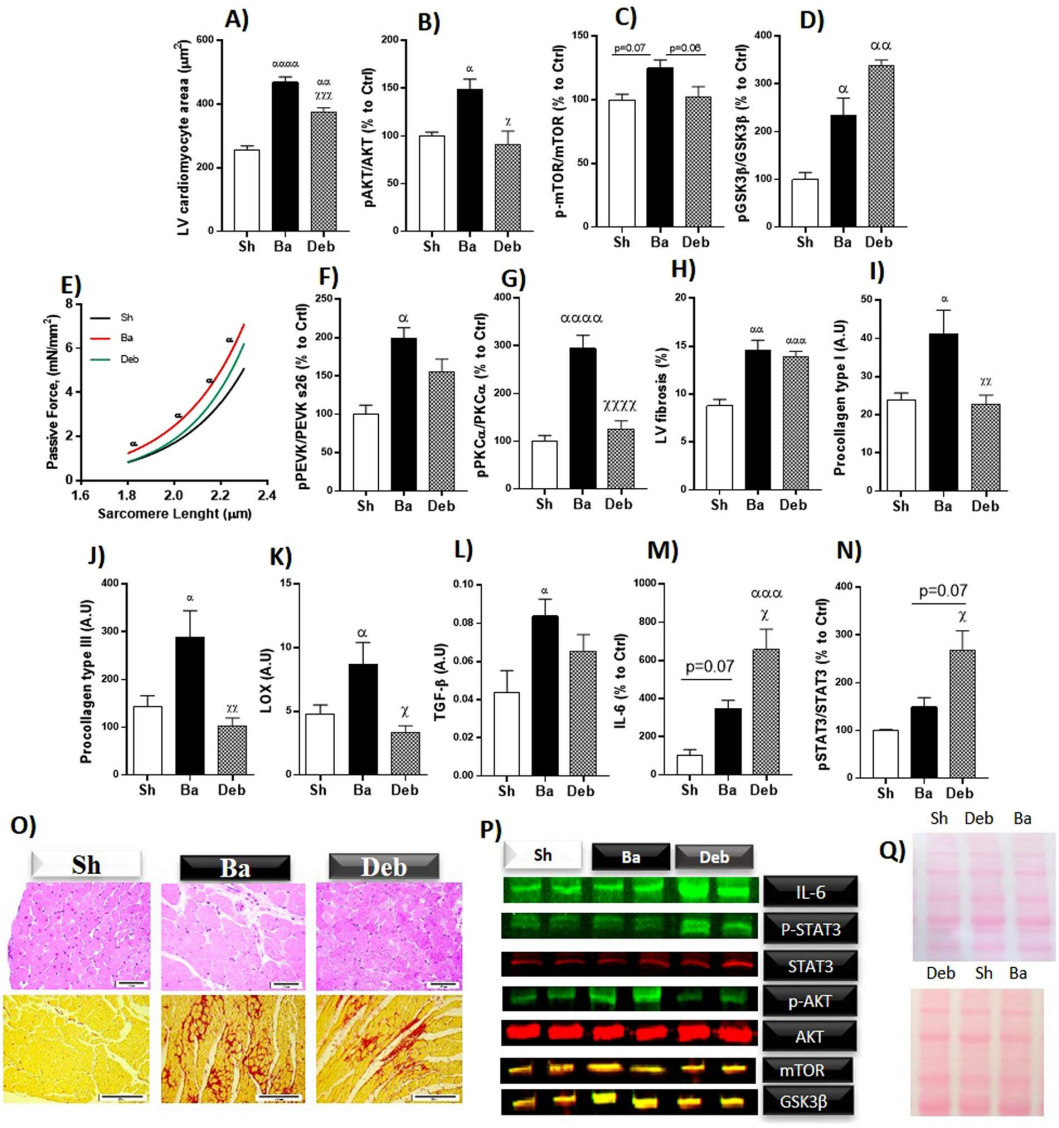
Effect of pressure overload on left ventricular hypertrophy, fibrosis, LV passive tension and extracellular matrix remodelling. (**A**) Cardiomyocytes area evaluated by histology; Western blot relative quantification of: (**B**) ratio of phosphorylated to total AKT; (**C**) ratio of phosphorylated to total mTOR; (**D**) ratio of phosphorylated to total glycogen synthase kinase 3 beta (GSK3β); (**E**) cardiomyocytes passive tension; (**F**) ratio of S26 phosphorylation to total Titin PEVK segment; (**G**) ratio of pPKCα to total PKCα phosphorylation; (**H**) myocardial fibrosis; (**I**) mRNA expression of procollagen type-I; (**J**) mRNA expression of procollagen type-III; (**K**) lysyl oxidase; (**L**) mRNA expression of *Transforming growth factor beta*, (**M**) Interleukin-6; (**N**) ratio of phospho to total Signal transducer and activator of transcription 3; (**O**) representative images of cardiomyocyte hypertrophy and myocardial fibrosis; (**P**) representative western blot lanes. For representative purposes the image was cropped and edited; (**Q**) representative membrane protein loading. n = 7 for each group. Values are mean ± SEM, Two-way ANOVA for passive tension, One-way ANOVA for the remaining data. Ba/Deb vs Sh: ^α^p < 0.05; ^αα^p < 0.01; ^ααα^p < 0.001; Deb vs Ba: ^χ^p < 0.05; ^χχ^p < 0.01.

Cardiomyocyte area increased in the Ba relative to the sham group and was attenuated in the Deb group (Fig. 1A,O). Accordingly, we observed an overactivation of pro-hypertrophic pathways as assessed by an increase of p-AKT/AKT, p-GSK3β/GSK3β and a trend of p-mTOR/mTOR to increase (Fig. 1B–D). In Deb rats, except for GSK3β which further increased, the activation of these hypertrophic pathways normalied (Fig. 1B–D,P).

When compared to sham, Ba rats displayed increased LV cardiomyocyte stiffness, as shown by a steeper passive tension versus sarcomere length (SL) relation (Fig. 1E). This stiffness was accompanied by an increase of titin phosphorylation at PEVK segments and PKCα phosphorylation (Fig. 1F,G). In the Deb group, cardiomyocyte stiffness was partially reversed since we observed a reduction of passive tension (at higher SL) and, accordingly, a decrease of PKCα and PEVK segment phosphorylation.

Myocardial fibrosis induced by aortic banding did not decrease after debanding (Fig. 1H), despite the normalisation of *procollagen type I* and *type III* and *Lox* expression (Fig. 1I–K). Regarding the proinflammatory and profibrotic pathway IL-6-STAT3 and *Tgf-β* we observed that, when compared to sham, banding animals showed an increase of *Tgf-β* in parallel with a slight, non-significant, increase of IL-6 and STAT3 activation (Fig. 1L–N). Interestingly, overload relief triggered a significant upregulation of IL-6-and STAT3 activation, despite the slight reduction in *Tgf-β* (Fig. 1L–N).

Considering the persistent LV cardiomyocyte hypertrophy and LV fibrosis, the over-activation of GSK3β, STAT3 and higher levels of IL-6 in the Deb group, we aimed at exploring the diversity of myocardial RR among the Deb group. Therefore, we divided Deb animals into two subgroups according to the content of fibrosis and LV mass regression (<30% LV mass reduction and >15% of fibrosis = Deb 1; >30% LV mass reduction and <15% of fibrosis = Deb 2). Interestingly, we found that, animals that presented a worse pattern of LV RR (Deb 1) displayed increased levels of STAT3 and GSK3β activation as well as overexpression of IL-6, TGF-β, Galectin and TIMP2 (Fig. S3).

### Left ventricle cardiomyocyte force measurements and diastolic calcium homeostasis

**Figure 2:**
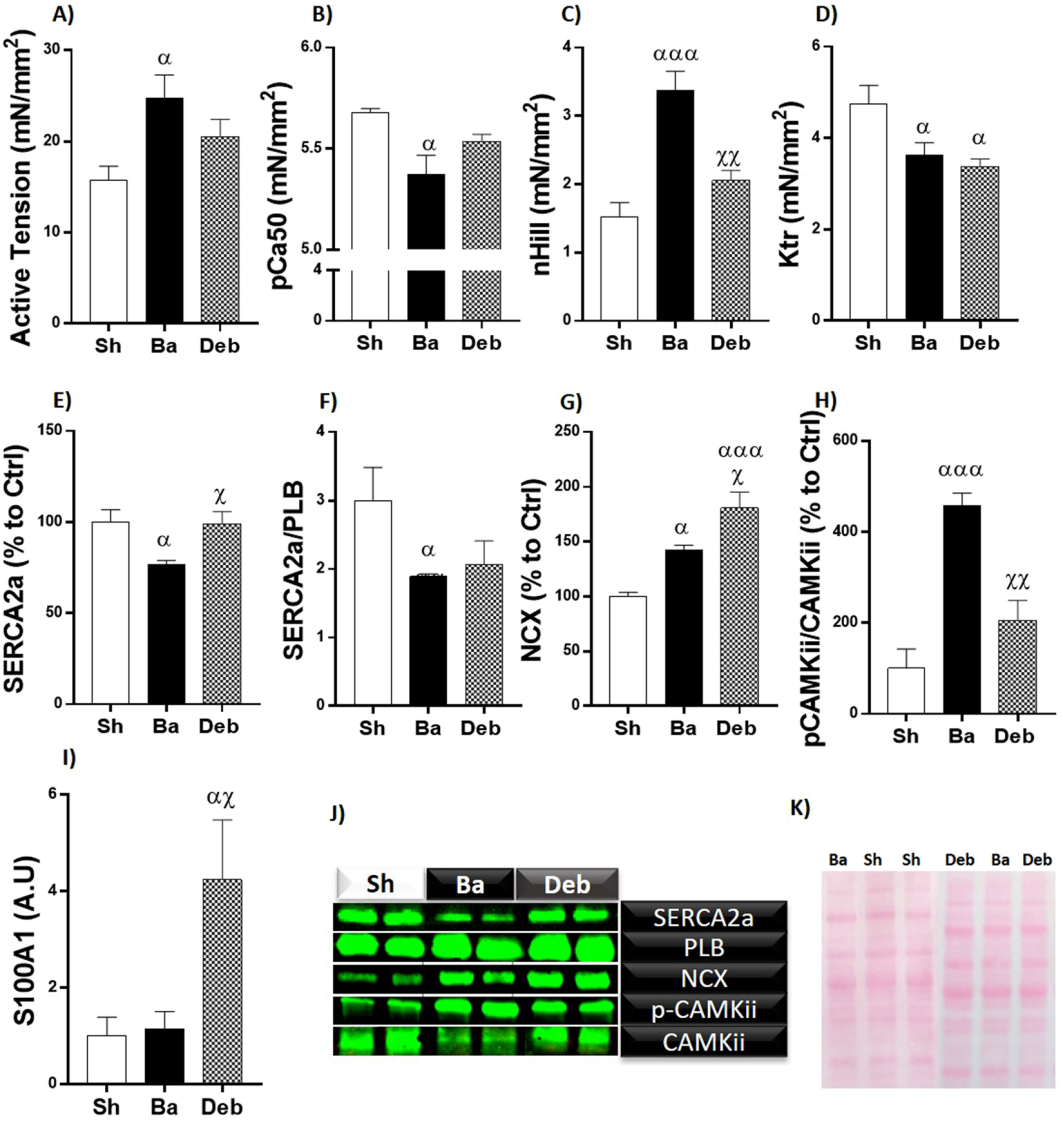
Effect of pressure overload on LV cardiomyocytes and calcium handling. (**A**) Cardiomyocytes active tension; (**B**) myofilaments calcium sensitivity; (**C**) *Hill*-coefficient; (**D**) the rate of tension redevelopment (Ktr); protein quantification of: (**E**) sarcoplasmic/endoplasmic reticulum Ca^2+^ATPase 2a (SERCA2a); (**F**) ratio of SERCA2a to phospholamban; (**G**) sodium-calcium exchanger; (**H**) ratio of phosphorylated to total Ca^2+^/calmodulin-dependent protein kinase (CAMKii); mRNA expression of calcium-binding protein A1 (S100A1); (**J**) representative western blot lanes. For representative purposes the image was cropped and edited; (**K**) representative membrane protein loading. Values are mean ± SEM. One Way-ANOVA: Ba/Deb vs Sh: ^α^p < 0.05; ^αα^p < 0.01; ^ααα^p < 0.001; Deb vs Ba: ^χ^p < 0.05; ^χχ^p < 0.01, ^χχχ^p < 0.001.

Cardiomyocytes from the Ba group presented increased active tension, calcium sensitivity (pC50), myofilament cooperativity (nHill) and a reduction of the rate constant of force redevelopment (Ktr, (Fig. 2A–D) compared to the sham group. In Deb animals, while nHill fully recovered, active tension and pCa50 decreased but did not normalise, while Ktr remained decreased (Fig. 2A–D). Regarding the expression of proteins associated with diastolic calcium homeostasis, banding animals presented reduced levels of SERCA2a and SERCA2a/PLB ratio and augmented NCX content. Inversely, phosphorylated CAMKii was significantly increased (Fig. 2E–H). In Deb rats, SERCA2a expression normalised but the same was not observed for SERCA2a/PLB (Fig. 2E,F). Additionally, we found CAMKii to be less active, as assessed by its decreased phosphorylation levels, and upregulation of *S100a1* after pressure-overload relief (Fig. 2H,I).

### RV structure and function

In Ba rats, right atrium dimensions increased while echocardiographic markers of systolic function, such as TAPSE and S′, decreased (Table 2). Regarding diastole, the velocity of E′ as well as E/A ratio decreased (Table 2). After afterload relief, some of these variables did not normalise completely, persisting significantly different from the sham group (Table 2). Together these data evidenced some degree of right-ventricle impairment triggered by aortic constriction.

**Table 2:** Right ventricle morphological and functional data.

	Sh (n = 7)	Ba (n = 10)	Deb (n = 8)
RAA (cm^2^)	0.207 ± 0.006	0.246 ± 0.009^αα^	0.254 ± 0.009^αα^
TAPSE (cm)	0.306 ± 0.009	0.230 ± 0.013^ααα^	0.257 ± 0.008^α^
S′ (ms)	0.065 ± 0.003	0.0499 ± 0.004^αα^	0.057 ± 0.003
E′ (ms)	0.064 ± 0.007	0.044 ± 0.005^α^	0.041 ± 0.003^α^
E (ms)	0.37 ± 0.02	0.42 ± 0.04	0.39 ± 0.03
A (ms)	0.40 ± 0.02	0.63 ± 0.05^αα^	0.58 ± 0.06^α^
E/A	0.95 ± 0.05	0.58 ± 0.06^αααα^	0.68 ± 0.05^αα^
Lungs/BW (g.Kg^−1^)	4.300 ± 0.117	4.821 ± 0.168^α^	4.316 ± 0.101^χ^
VO_2_max (ml.min^−1^.kg^0.75^)	33.3 ± 1.0	29.2 ± 0.7^αα^	31.3 ± 0.8

**RV**, Right ventricle; **RAA**, Right atrium area; **TAPSE**, tricuspid annular plane systolic excursion; **S′**, Peak systolic annular velocity; E**′**, wave velocity of tissue Doppler at the lateral tricuspid annulus; **E**, peak of pulse Doppler wave of early tricuspid flow velocity**; A**, peak of pulse Doppler wave of late tricuspid flow velocity**; E/A**, ratio between peak E and A waves; **BW**, body weight; **VO**_**2**_**max** maximum rate of oxygen consumption. Values are mean ± SEM. One Way-ANOVA, **Ba/Deb vs Sh**: ^α^p < 0.05; ^αα^p < 0.01; ^ααα^p < 0.001; **Deb vs Ba**: ^χ^p < 0.05; ^χχ^p < 0.01, ^χχχ^p < 0.001.

Lung weight increased in Ba animals evidencing pulmonary congestion. Effort intolerance was evidenced by lower VO_2_max in rats with pressure-overload but normalised after afterload relief. Interestingly VO_2_max correlates negatively with important LV functional and structural parameters, such as LV mass, LAA and E/e′ (Fig. S4).

In Deb rats, VO_2_max, E and S′ wave decreased to intermediate values between banding and sham group (Table 2).

### Right cardiomyocyte hypertrophy and fibrosis

**Figure 3:**
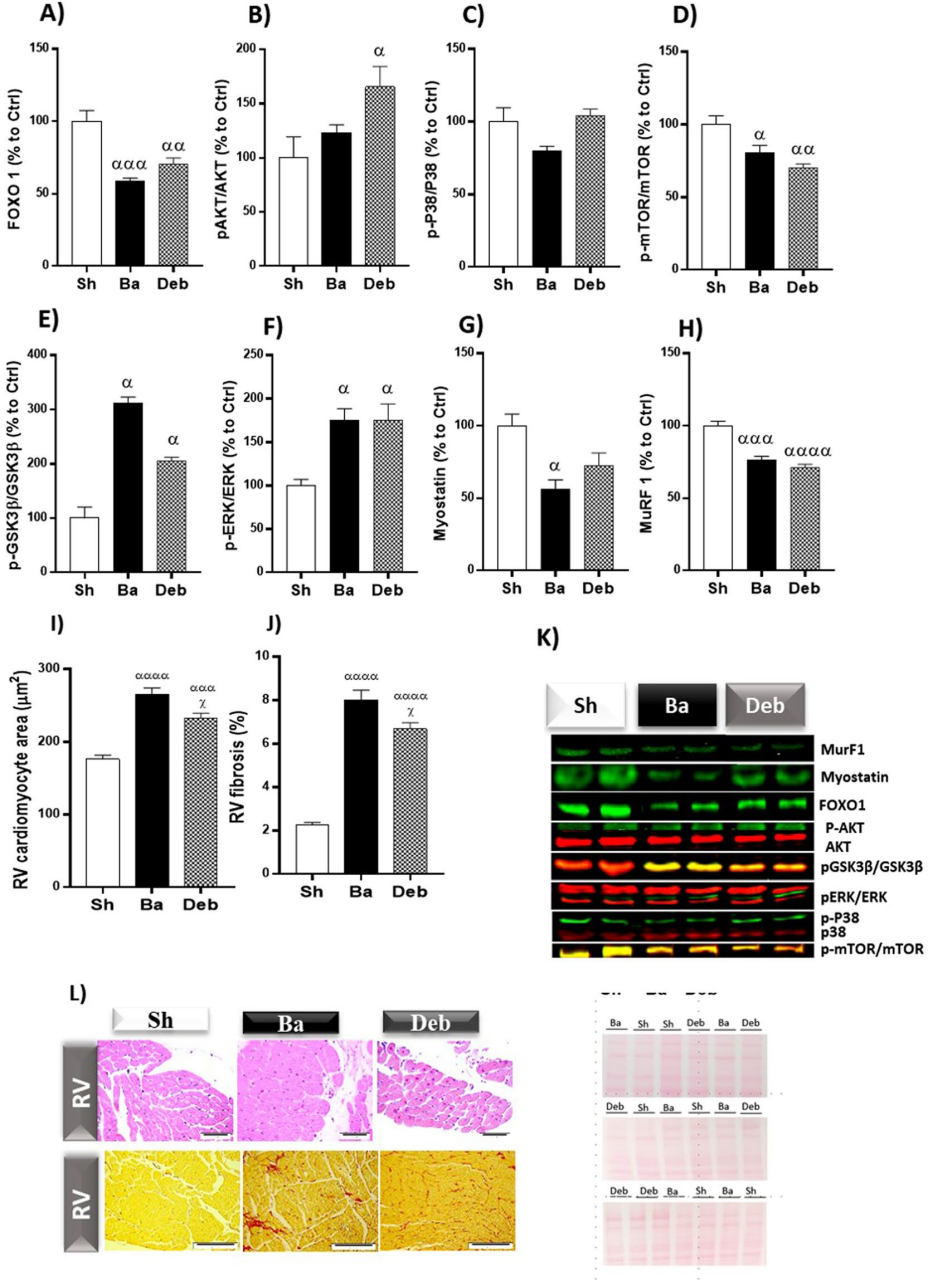
Effect of pressure overload on RV hypertrophy and fibrosis. (**A**) Foxo transcription factor 1; (**B**) ratio of phosphorylated to total protein kinase B or AKT; (**C**) ratio of phosphorylated to total P38 mitogen-activated protein kinase; (**D**) ratio of phosphorylated to total mammalian target of rapamycin (mTOR); (**E**) ratio of phosphorylated to total glycogen synthase kinase 3 beta (GSK3β); (**F**) ratio of phosphorylated to total extracellular signal–regulated kinase (ERK); (**G**) Myostatin; (**H**) Muscle-specific RING finger protein-1 (MuRF1); (**I**) representative western blot lanes (for representative purposes the image was cropped and edited) representative membrane protein loading. n = 5 for each group. Values are mean ± SEM, One-way ANOVA. Ba/Deb vs Sh: ^α^p < 0.05; ^αα^p < 0.01; ^ααα^p < 0.001; Deb vs Ba: ^χ^p < 0.05; ^χχχ^p < 0.001.

In the RV, while chronic pressure-overload induced a decrease of transcription factor (FOXO1) and mTOR, P38 activation and AKT were not significantly altered (Fig. 3A–D), but the latter was overactivated by pressure-overload relief (Fig. 3B). While the anti-hypertrophic activity of GSK3β induced by banding was slightly attenuated after debanding (Fig. 3E), ERK phosphorylation remained higher compared to sham (Fig. 3F). Myostatin and muRF1, were downregulated in banding, but the former was slightly increased in debanding (Fig. 3G,H).

Banding group showed increased RV cardiomyocyte area and fibrosis (Fig. 3I,J). These values decreased in debanding compared to banding but did not normalise to sham values.

### Right ventricle cardiomyocyte force measurements and calcium homeostasis

**Figure 4:**
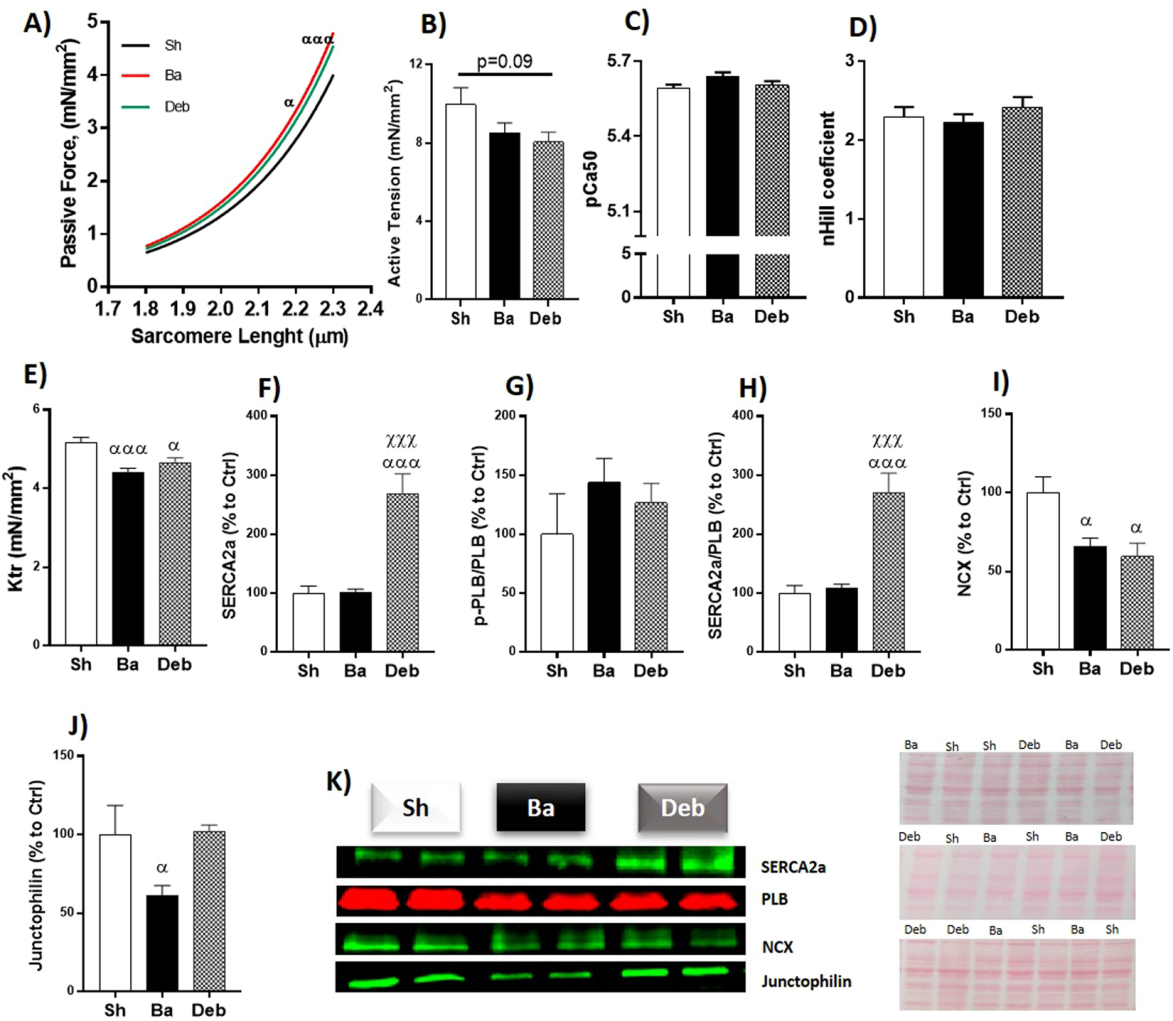
Effect of pressure overload on RV cardiomyocytes and calcium handling. (**A**) cardiomyocytes passive tension; (**B**) cardiomyocytes active tension; (**C**) myofilaments calcium sensitivity; (**D**) *Hill*-coefficient; (**E**) the rate of tension redevelopment (Ktr); Protein quantification of: (**F**) sarcoplasmic/endoplasmic reticulum Ca^2+^ATPase 2a (SERCA2a); (**G**) ratio of phospholamban phosphorylated (p-PLB) to total (PLB); (**H**) ratio of SERCA2a to phospholamban; (**I**) sodium-calcium exchanger (NCX); (**I**) Junctophilin; (**L**) representative western blot lanes (for representative purposes the image was cropped and edited) representative membrane protein loading. n = 5 for each group. Values are mean ± SEM, One-way ANOVA. Ba/Deb vs Sh: ^α^p < 0.05; ^αα^p < 0.01; ^ααα^p < 0.001; Deb vs Ba: ^χ^p < 0.05; ^χχχ^p < 0.001.

When compared to sham, Ba rats displayed increased RV cardiomyocyte stiffness (as shown by a steeper passive tension versus SL relation) and decreased Ktr (Fig. 4A,E).

Aortic constriction relief slightly increased Ktr towards normalisation and induced a trend to cardiomyocytes’ active tension to decrease when compared to sham group (Fig. 4B).

Regarding the expression of proteins associated with calcium homeostasis, Ba animals presented reduced content of NCX and Junctophilin (Fig. 4J,K). After debanding SERCA2a and Junctophilin levels increased (Fig. 4F,K) while NCX remained lower compared to sham (Fig. 4B).

### Pulmonary arteries thickness

**Figure 5:**
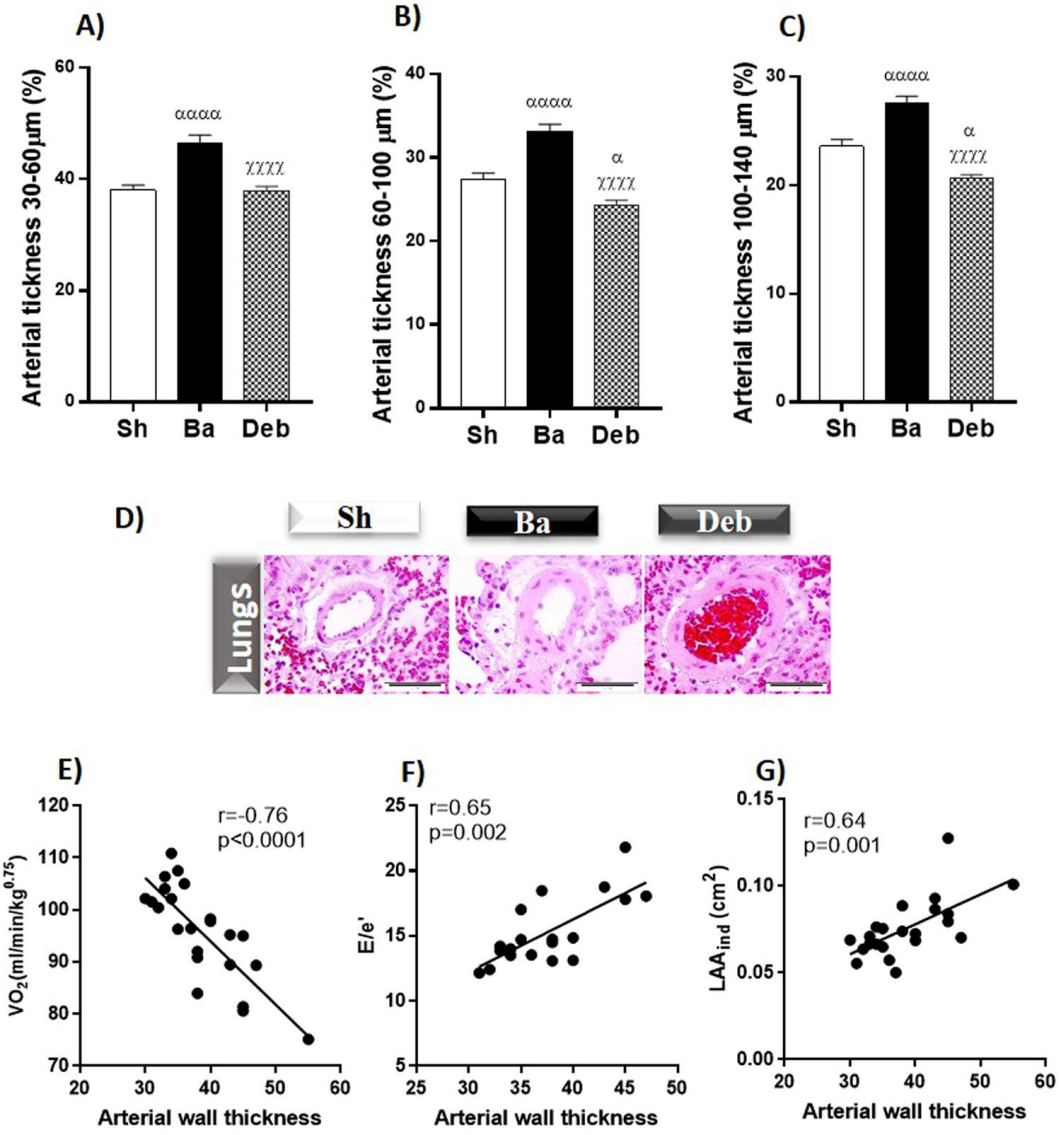
Effect of pressure overload on lung arteries thickness. (**A**–**C**) Pulmonary arterial thickness (**D**) representative images of histological lungs. Correlations between arterial wall thickness and: (**E**) maximum rate of oxygen consumption (VO_2_max); (**F**) ratio between: (**E**), peak of pulse Doppler wave of early mitral flow velocity and E′, wave velocity of tissue Doppler at the lateral mitral annulus; (**E**/e′) and (**G**) Indexed left ventricle area (LAA_ind_). n = 8 for each group. Values are mean ± SEM, One-way ANOVA Ba/Deb vs Sh: ^α^p < 0.05; ^αα^p < 0.01; ^ααα^p < 0.001; Deb vs Ba: ^χ^p < 0.05; ^χχχ^p < 0.001.

The thickness of pulmonary arteries induced by aortic constriction normalized completely after debanding. Interestingly, the thickness of the pulmonary arteries correlates negatively with exercise capacity and positively with E/e′ and indexed LAA (Fig. 5G,H,I).

## Discussion

The underlying mechanisms associated with incomplete myocardial RR need to be clarified to successfully potentiate myocardial recovery after AVR with a special emphasis on RV involvement. Banding rats resemble AS patients by presenting LV concentric hypertrophy, DD (alterations of the pattern of ventricular filling and myocardial relaxation), pulmonary congestion and effort intolerance. Besides LV alteration, aortic constriction-induced several RV abnormalities.

Two weeks after overload relief, reduction of aortic velocity trigger reverse remodelling and cardiac function amelioration (more evident on left heart). On the LV, myocardial hypertrophy, fibrosis and atrium area enlargement remained altered after pressure-overload relief. In addition to sustained LV fibrosis, the incomplete reversion of titin PEVK phosphorylation contributed to the persistent myocardial stiffness in debanding rats. The improvement of relaxation during RR is probably related to the reduced CAMKii activity, and increased Serca2a and *S100a1* expression that potentially restrains calcium leak by the ryanodine receptor (RyR) and improves calcium reuptake by the sarcoplasmic reticulum (SR). Importantly, this model presents significant RV abnormalities. Some of these abnormalities do not revert during RR, such as hypertrophy, fibrosis, right-atria enlargement and systolic and diastolic markers of cardiac dysfunction. At the cell level, and similarly to the LV, RV cardiomyocytes from banding rats were stiffer and showed a reduced rate of force redevelopment (Ktr). This manuscript provides novel insights on LV and RV changes secondary to chronic pressure overload and highlights the involvement of both ventricles in chronic pressure overload conditions imposed on the LV, such as in the case of aortic stenosis.

**Figure 6:**
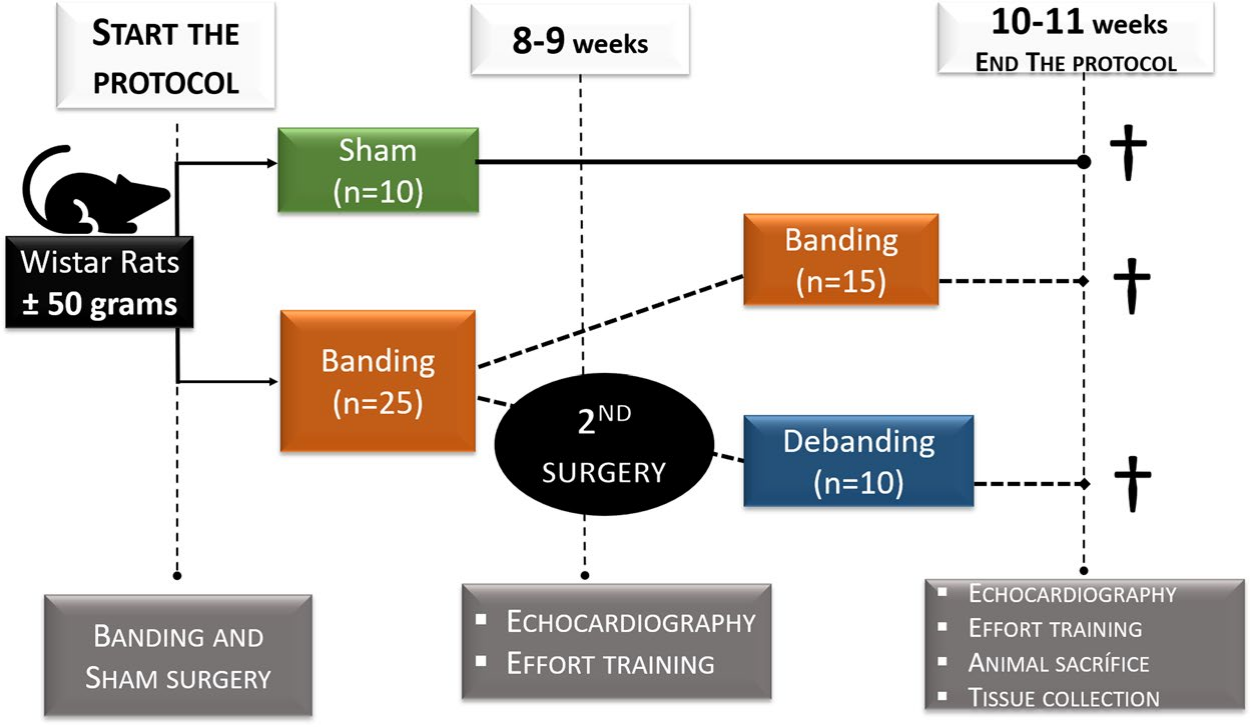
Experimental protocol design.

Diastolic dysfunction is frequently found in AS patients with normal systolic performance. In severe AS with preserved EF, symptomatic patients show impaired diastolic function and atrial dilatation^17^. Therefore, we aimed to detect early changes that might represent important time points for therapeutic interventions aiming to reverse the progression of the disease. Traditionally AVR has been recommended only after the onset of HF symptoms and a mean gradient higher than 40 mmHg^18^. However, recent evidence suggests that AVR outcome can be improved if performed earlier, as soon as diastolic function starts to decline^19,20^. Thus, in our aortic-banding model (Fig. 6), we monitored and studied myocardial remodelling as soon as LV DD and LV concentric hypertrophy became evident at echocardiographic level (Fig. S1). The mechanisms by which pressure-overload induces LV hypertrophy are, among others, increased AKT, mTOR, and GSK3β phosphorylation^21^. In our study, activation of AKT pro-hypertrophic signalling predicts the left ventricular adaptive response aiming to normalise wall stress and compensate for the increased afterload. Myocardial stiffness and impaired relaxation are hallmarks of DD and usually result from LV concentric hypertrophy induced by pressure overload^17,22^. Myocardial stiffness is strongly determined by alterations in the extracellular matrix, such as myocardial fibrosis, and by changes in cardiomyocyte myofilamentary proteins, mainly titin, a major contributor to cardiomyocyte passive tension. In Ba rats, increased collagen deposition, the over-expression of the main enzyme responsible for collagen cross-linking (i.e. LOX), and the hyperphosphorylation of Titin S26-PEVK segment contributed to increased LV myocardial stiffness by augmenting myocardial fibrosis, collagen cross-linking and cardiomyocyte passive tension, respectively. The LV delayed relaxation in Ba rats can be attributed to the reduction of SERCA2a/PLB, hindering Ca^2+^ reuptake to the SR.

In the hypertrophic and hypercontractile myocardium of Ba rats, active tension, calcium sensitivity and cooperativity increased while Ktr decreased. Myocardial hypercontractility occurs in parallel with changes in myofilamentary proteins that increase force generation at a given calcium concentration^23–25^. Furthermore, it has been postulated that alterations in myofilamentary proteins are important for the development of familial hypertrophic cardiomyopathy^26^.

Importantly, in agreement with a previous study^27^, when the RV is overloaded, it develops several abnormalities, such as hypertrophy and deterioration of systolic and diastolic function. Similar results were recently obtained in patients who underwent AVR, and no significant change in RV function was observed^28^. Calcium overload and impaired ventricular relaxation can promote pulmonary congestion^29^ as observed in our banding animals and strengthening our positive correlation data between arterial wall thickness and some important parameters of diastolic dysfunction, namely E/e′ and LAA. Therefore, pulmonary vascular remodelling can be a consequence of the sustained increase in pulmonary venous pressure^30^. From the epidemiologic-standpoint, pulmonary hypertension is frequently present in AS patients and the underlying reason may be attributed to DD^31^. Interestingly in our banding rats, pulmonary arterial wall thickness correlates negatively with VO_2_ max and positively with DD, showing an interplay between these parameters. In the AS clinical scenario, pulmonary hypertension and DD can affect the quality of life of these patients by limiting their functional capacity, mainly during exercise^32,33^.

Aortic constriction triggered RV cardiomyocyte hypertrophy and downregulation or inactivity of anti-hypertrophic proteins such as muRF1, myostatin and GSK3β and activation of the pro-hypertrophic ERK. Despite the unquestionable involvement of these proteins on hypertrophic response to afterload, the activation of the well-recognised pro-hypertrophic agent, mTOR, decreased, AKT was unaltered, and the transcription factor 1 (FOXO1) decreased in the RV of Ba rats. Thus, in RV cardiomyocytes’ hypertrophy is probably mediated by activation of GSK3β, ERK, muRF1, myostatin and FOXO1.

In addition to RV myofilamentary passive tension in the banding group, myocardial fibrosis further contributed to RV stiffness. Similar to LV, RV cardiomyocytes showed a reduction in the rate of tension development with no significant changes in active tension.

Notwithstanding the myocardial remodelling changes imposed by chronic pressure overload, our main goal was to describe the changes during RR. This is quite pertinent since only a few studies describe the changes induced by aortic debanding at an early stage of the disease^15,16,34–36^ and most of the available information on RR derives from LVAD studies. However, RR-induced by LVAD is imposed on a totally different phenotype characterised by dilated hypertrophy and systolic dysfunction^37–46^. This is markedly contrasting to the concentric hypertrophy and DD patent in our animal model that was designed to resemble AVR-induced RR and to assess the early events of myocardial RR.

Clinically, the classical parameters to evaluate the success of RR are EF, LV end-diastolic and end-systolic volumes, LV mass and, more recently, diastolic function^47^. After debanding, the pressure relief allowed the myocardium to remodel as denoted by the partial recovery of LV mass, normalisation of LV volumes and amelioration of LV diastolic dysfunction. Although, RV abnormalities induced by LV pressure overload prevail during myocardium RR, independently of pulmonary edema reduction. This data shows that after aortic pressure relief, the RV takes longer than the LV to recover. Recent studies has shown RV function deterioration after aortic valve replacement^14^. The mechanism that underlies RV dysfunction post-surgery were not addressed nor discussed in the existing literature. Herein, we were able to describe major changes at cardiomyocyte, myofilaments and calcium levels, adding relevant information to the state of the art.

We expected that, due to the early nature of our intervention, Deb animals would normalise hypertrophy by reducing AKT pro-hypertrophic signalling pathway, namely phosphorylation of GSK3β and mTOR. However, despite the reduction of AKT and mTOR activation, LV mass and GSK3β phosphorylation remained increased in Deb rats compared to Sham, rats. Since the anti-hypertrophic effects of GSK3β can be blocked by kinases other than AKT or CAMKii^48,49^, we speculated that these kinases (not assessed in our study) might have contributed for the persistent GSK3β activation after overload relief.

During RR, RV cardiomyocytes’ hypertrophy and fibrosis remained high. The downregulation or decreased activity of anti-hypertrophic proteins such as myostatin, GSK3β or FOXO1 could underlie the persistent hypertrophy. Nevertheless, mTOR activation remained decrease and AKT and ERK increased compared to sham, highlighting the complex interaction between pro- and anti-hypertrophic pathways.

In addition to LV hypertrophy, myocardial stiffness represents an important postoperative clinical marker to determine the RR outcomes after AVR as it strongly associates with DD^50^. Of note, after AVR, the survival rate is lower in patients with severe fibrosis^47^. Myocardial fibrosis results from an excessive collagen deposition due to an imbalance in its turnover, and to increased collagen cross-linking^51,52^. Although in our study collagen deposition was not modified after overload relief in none of the ventricles, we recognise that a longer period could be necessary to report a significant decrease of myocardial fibrosis. On the other hand, the interplay between fibrosis and inflammation can regulate extracellular matrix remodelling. In fact, IL-6-induced STAT3 activation can promote fibrosis, at least indirectly, by enhancing TGF-β signalling^27^ or TIMP-1 expression^53^. Thus, the activation of the IL6-STAT3 proinflammatory and profibrotic pathways could contribute to the maintenance of myocardial fibrosis in Deb rats. Notwithstanding, rats that showed a worse pattern of reverse remodelling present increased activation of proinflammatory and profibrotic pathways (e.g. IL-6-STAT3, TGF-β, etc).

Apart from the extracellular matrix contribution, titin and other myofilamentary proteins also impact myocardial stiffness. Indeed, relatively few information is available about titin changes in the context of AVR-induced LV RR^54^. In LV from debanding group, we observed a non-significant attenuation of passive tension that was, at least in part, consequence of S26-PEVK segment phosphorylation, decreasing PEVK persistence length, and increasing titin-based passive force^55^. Intriguingly, when compared to Sh rats, PKCα normalised and CAMKii is still upregulated. PKCα and CAMKii have opposite effects on titin passive tension, while the former increases, the latter decreases stiffness^55–58^. Thus, we propose that the balance of both kinases could contribute to the titin based-passive tension in Deb group. Indeed, titin-derived stiffness results from a fine balance between kinases activity in over 2000 residues that compose the PEVK and others titin-segments, whose numerous potential phosphorylation sites still await validation^55,56,58,59^.

Regarding myofilamentary changes during RR, we have shown that calcium sensitivity decreased, the myocardium contractility was reduced, and myofilaments active tension and cooperativity was attenuated. In parallel, Ktr sustained decrease meaning that the rate of crossbridges recycling was still reduced, maybe due to a shift towards β-myosin heavy chain, which impairs cross-bridge cycling^12^. Indeed, Bjørnstad *et al*. demonstrated the upregulation of β-myosin heavy chain to persist after mice aortic debanding^16^. In our study, if the decrease of Ktr is detrimental or beneficial in LV remodelling or LV RR is still unknown. However, the translation/clinical importance of this finding is relative since humans express predominantly the β-myosin heavy chain isoform in ventricular myocytes as opposed to the α-myosin heavy chain isoform that predominates in rodents^60^.

Finally, another important contributor for DD in AS is myocardial active relaxation associated with calcium-handling. Two-weeks of RR triggered myocardial relaxation to recover to sham values possibly a consequence of the decrease in CaMKii phosphorylation or the up-regulation of calcium-binding protein S100A1. These changes can prevent diastolic SR calcium release and calcium leak^24,29^ and thus contribute to the improved relaxation observed during RR.

### Limitations

This study aimed to provide an overview of the major biventricular changes taking place during myocardial reverse remodelling. Thus, this study represents an initial approach to describe important signalling pathways related to cardiomyocytes’ myofilaments, calcium-handling, hypertrophic and inflammatory signalling pathways as well as to the extracellular matrix in an early stage of AS progression. Thus, its nature is essentially descriptive, opening novel perspectives for the scientific and medical community to carry out future studies, for instance, focusing on the *in vitro* modulation of myofilamentary proteins function or targeting right ventricular changes.

## Conclusion

This work describes several mechanisms underlying cardiac remodeling and RR and highlights that this model nicely recreates important phenotypic features (myocardial hypertrophy and extracellular matrix remodelling, calcium handling and myofilaments’ changes) of chronic pressure overload and its relief, respectively. These rats demonstrate LV concentric hypertrophy and DD, RV dysfunction, remodeling of pulmonary arteries and, importantly, exercise intolerance. After debanding, the normalisation of cardiac function was not paralleled by the reversion of alterations in cardiac structure, myocardial fibrosis and inflammation. Importantly, banding-induced changes in RV function and structure persisted after debanding, despite the complete reversal of pulmonary vascular remodelling, demonstrating a possible masked effect of RV dysfunction.

## Methods

### Experimental animal model

#### Ascending Aortic Banding and Debanding

Young male Wistar rats (±50 g) were anasthetized by inhalation of 8% sevoflurane, orotracheally intubated and mechanically ventilated (TOPO Small Animal Ventilator, Kent Scientific Inc). Anaesthesia was maintained with sevoflurane (2.5–3%). The animals were placed in right-lateral decubitus on a heating pad. After a small incision between the 2^nd^ and 3^rd^ intercostal space aortic banding was surgically made with a 22-gauge blunt needle placed parallel to the aorta. A ligature (5–0; polypropylene) was firmly tied around both, and the needle was subsequently removed (Banding group, Ba, n = 15). In the sham group, the suture was kept loose (Sh, n = 10). The thorax was closed and the animal allowed to recovered with proper analgesia (Buprenorphine, 0.05 mg.Kg^−1^, twice daily). The cardiac function and structure were followed by echocardiography. When LV mass was ≥25%, and DD was observed (8–9 weeks) (Fig. 6), a second surgery was made in 10 of the Ba animals to remove the suture, debanding group (Deb, n = 10). Animals were sacrificed 2 weeks later. Experiments were performed according to the Guide for the Care and Use of Laboratory Animals published by the NIH (NIH Publication no. 85–23, revised 2011) and with the Portuguese law of animal welfare (DL 129/92, DL 197/96; P 1131/97). The project was approved by the ethical committee of the Faculty of Medicine of the University of Porto and the Portuguese Foundation for Science and Technology and certified by the Portuguese National Authority for Animal Health (PTDC/ DTP-FTO/0130/2012; 0421/000/000/2013).

### Exercise tolerance, Echocardiographic and haemodynamic evaluation

Aerobic capacity was evaluated on a treadmill chamber coupled to a gas analyser (LE8700C and LE405, Panlab Harvard Apparatus^®^). The treadmill was tilted to 10°. The adaptation was carried out at a speed of 15 cm.s^−1^ for 3 minutes. The maximum stress test started at a speed of 30 cm.s^−1^, with increments of 5 cm.s^−1^ every minute until the animals reached maximal aerobic capacity (VO_2_max).

For assessing *in vivo* cardiac function, rats were anaesthetized as mentioned above. A linear 15 MHz probe (Sequoia 15L8W) was used for the echocardiographic studies. M-mode was used to determine systolic and diastolic wall thickness and cavity dimensions. Ejection fraction, fractional shortening and LV mass were calculated as previously described^61^.

Mitral flow velocity tracings were obtained with pulsed-wave Doppler, peak systolic tissue velocity and E′ were measured with tissue Doppler. Atrial dimensions and TAPSE were measured in the four-chamber view. Acquisitions were made using an echocardiograph (Siemens Acuson Sequoia C512). Recordings were averaged from three consecutive heartbeats.

For the haemodynamic evaluation, femoral vein was catheterized for fluid administration, and a left lateral thoracotomy exposed the heart allowing for the insertion of a catheter into the LV (SPR-*847*. 1.4 F, Millar instruments. Baseline and inferior vena cava occlusion recordings were obtained with ventilation suspended at end-expiration. Data was continuously acquired (MPVS 300, Millar Instruments) at 1000 Hz (ML880 PowerLab 16/30, Millar Instruments) and analysed off-line by PVAN software (Millar Instruments). Parallel conductance was computed after hypertonic saline bolus. In the end, while anesthetised, the animals were sacrificed by exsanguination, the tissues were collected, weighed and properly stored for molecular and functional studies.

### Histology

The right ventricle (RV), LV and lungs were fixed in formalin, dehydrated in ethanol, cleared in xylol and impregnated in paraffin. Five-micrometer slides were dewaxed, rehydrated, and stained with

Haematoxylin-Eosin (HE), to assess cardiomyocyte area, or Picrosirius Red, to assess myocardial fibrosis, and finally mounted with Entellan®. An optic microscope (Leitz Wetzlar – Dialux 20, Wetzlar, Germany), equipped with a photographic camera (Olympus XC30, Tokyo, Japan) was used to visualise and photograph the histological preparations. The area of 60 cardiomyocytes per animal was measured using Cell^B software (Olympus). To calculate the area of fibrosis eight fields per animal were photographed and analysed with Image-Pro Pus 6 software (Media Cybernetics, Rockville, USA). To assess pulmonary arteries remodelling, HE staining was used, and artery medial wall thickness (WT) was expressed as follows: %WT = [(Medial wall thickness × 2)/Arterial external diameter] × 100. Analysis was performed in a blind mode. We attributed a code to each sample and the correspondence to the experimental group was done after analysis of the results.

### Force measurements in isolated cardiomyocytes

After defrosted in 2.5 mL of calcium-free relaxing solution LV samples were mechanically disrupted, and the cardiomyocyte suspension incubated with 0.5% Triton X-100 for 5 minutes at room temperature to permeabilize the membranes. To remove the detergent, the cells were washed with relax solution and centrifuged (1500 rpm, 1 min, 4 °C) 4 to 5 times. Force measurements were performed as described previously^24^ using a force transducer from Aurora Scientific Inc. (Model 403A) and a length controller (Model 315C-I). Briefly, a passive tension-length relation was done stretching the cell until a SL of 2.2 µm. Maximal activation at pCa 4.5 was used to calculate maximal calcium-activated isometric force (Total tension, Tt) and the slack test (the cell was shortened for 1 ms to 80% of its original length) allowed to measure the rate of force-redevelopment (Ktr). A relaxing solution pCa 9.0 was used to determine passive tension (Tp). In the end, the cell was activated (pCa = 4.5) to access the cardiomyocyte functional stability and integrity. Lastly, the dimensions of the cell were measured, and the force values were normalized to the cardiomyocyte cross-sectional area. Data acquisition was made by ASI 600 A program with a sampling frequency of 2 KHz.

### Protein analysis by Western Blot

Tissue was homogenized in RIPA buffer (150 mM NaCl, 1.0% IGEPAL^®^ CA-630, 0.5% sodium deoxycholate, 0.1% SDS, 50 mM Tris, pH 8.0; Sigma-Aldrich, R0278,) and protein concentration was determined based on the method of Bradford (Bio-Rad Protein Assay, 500–0006). Twenty micrograms (20 μg) of protein of each sample was prepared with Laemmli buffer (1 M Tris-HCl pH 6.8, 10% SDS, 20% glycerol, 0.004% bromophenol blue, 20% 2-mercaptoethanol) randomly loaded and separated by SDS-PAGE (Mini-PROTEAN Tetra Cell, Bio-Rad). After transferring to a nitrocellulose membrane (Bio-Rad 1620115 and 1620112) and blocking with 5% BSA (w/v; A2058, Sigma-Aldrich) in TBS-0.5%Tween (Tris-buffered saline-Tween 20) the membranes were cut and incubated with primary antibodies overnight at 4 °C. NCX (Santa Cruz,sc-32881), AKT (Cell Signalling, 9272), p-AKT (Cell Signalling, 9271), Junctophilin-2 (Santa Cruz, sc-377086), TIMP-1 (abcam ab38978), TIMP-2 (abcam ab1828), Galectin-3 (abcam, ab2785), MMP-2 (abcam 37150), MMP-9 (abcam ab38898), mTOR (Cell Signalling, 2972), p-mTOR (Cell Signalling, 2971), GSK3β (Cell Signalling, 9315), p-GSK3β (Cell Signalling, 9331), PKC (Abcam, ab32376) and p-PKC (abcam ab23513); IL-6 (Cell Signalling, 12153) Stat3 (Cell Signalling, 9132) p-Stat3 (Cell Signalling, 9131) SERCA2a (Cell Signalling, 4388); PLB (Thermo-Fisher, MA3–922), p-PLB (cell signalling, 8496) Camkii (SantaCruz, SC-5306) and p-Camkii (SantaCruz, SC-32289). Finally, membranes were incubated for an hour at room temperature with secondary antibodies (IRDye 800CW and IRDye 680LT, LI-COR). The signal was detected by an image acquisition system (Odyssey Infrared Imaging System LI-COR Biosciences at 700 or 800 nm) and the image acquired prior to signal saturation. The signal was normalised to total protein density (ponceauS (Sigma-Aldrich P7170) as previously described^62^.

For titin analyses, cardiac muscle tissue was homogenized in modified *Laemmli buffer*^63^ and 1.8% agarose gels were used to separate the proteins by vertical SDS–agarose electrophoresis (run at 15 mA per gel for 15 h) (Minigel-Twin, Biometra). To determined titin isoforms the gels were stained with Coomassie brilliant blue. For titin Western blotting (WB) LV samples were run on 1.8% agarose gels and transferred to PVDF membrane (Millipore, Immobilon®-FL Cat. No. IPFL00010 PVDF-membrane by semi-dry Western blot technique using the Biorad turbo blot system (1.5 A for 25 min with 20 V). The blots were stained with Ponceau S solution (Sigma-Aldrich P7170) to visualise total transferred protein. The blots were then probed with rabbit polyclonal antibodies against titin’s pS26 (GenScript, 1:1000) and pS170 (Genscript, 1:250). Bands were visualised using the LAS-4000 Image Reader (Fuji Science Imaging Systems, Stamford, Connecticut). Densitometry was performed using Multi Gauge version 3.2 software (Fuji Science Imaging Systems).

### Real-time quantitative Polymerase Chain Reaction (RT-qPCR)

For gene expression analyses, RNA was extracted with TriPure (Roche). RT-PCR was performed with total RNA, followed by real-time PCR analyses using the SYBR Green method in a StepOne Plus, Applied Biosystems. For the animal studies, results are relative to the mean obtained for the SHAM group (set as arbitrary units) and normalised for *18S*. Specific PCR primer pairs for the studied genes were *18S*, *Procollagen-type-I alpha1* and *Type-III aplha1*, *lysyl oxidase* (*Lox*), *transforming growth factor beta* (*Tgf-β*) and *calcium-binding protein A1* (*S100a1*).

### Statistical analysis

Results are expressed as mean ± SEM. Statistical analysis was performed using GraphPad Prism software. Shapiro-Wilk was used to assess a parametric distribution. Comparisons were performed by one or two-Way ANOVA and appropriate post-hoc tests were used. The probability values < 0.05 were considered significant.

## Supplementary information

Supplements SREP-18-36653A
