# Supplementary material for: Characterization of biventricular alterations in myocardial (reverse) remodelling in aortic banding-induced chronic pressure overload
Source: Sci Rep. 2019 Feb 27;9:2956. doi: 10.1038/s41598-019-39581-9 (PMC6393473; doi:10.1038/s41598-019-39581-9)

## Supplementary information

**Characterization of biventricular alterations in myocardial (reverse) remodelling in aortic banding-induced chronic pressure overload**

Daniela Miranda-Silva^1α^, Patrícia Gonçalves-Rodrigues^1α^, João Almeida-Coelho^1^, Nazha Hamdani^2^, Tânia Lima^1^, Glória Conceição^1^, Cláudia Sousa-Mendes^1^, Cláudia-Moura^1^, Arantxa González^4,5^, Javier Díez^4,5^, Wolfgang A. Linke^3^, Adelino Leite-Moreira^1^, Inês Falcão-Pires^1*^

**^1^**Dept of Surgery and Physiology, University of Porto, Porto, Portugal; **^2^** Dept of Systems Physiology, Ruhr University, Bochum, Germany; **^3^**Institute of Physiology II, University of Muenster, Muenster, Germany,**^4^**Program of Cardiovascular Diseases, Centre for Applied Medical Research, University of Navarra and CIBERCV, Pamplona, Spain; ^5^Department of Cardiology and Cardiac Surgery and Department of Nephrology, University of Navarra Clinic, Pamplona, Spain.

^α^both authors contributed equally

***Address correspondence to**:

Dr. Inês Falcão Pires

Department of Surgery and Physiology

Faculty of Medicine, Universidade do Porto

Prof. Hernâni Monteiro 4200-319 Porto, Portugal

Tel: [+351 220 426 805](tel:+351%2022%20042%206805)

Fax: [+351 225 513 646](tel:+351%2022%20551%203646)

[ipires@med.up.pt](mailto:ipires@med.up.pt).

| **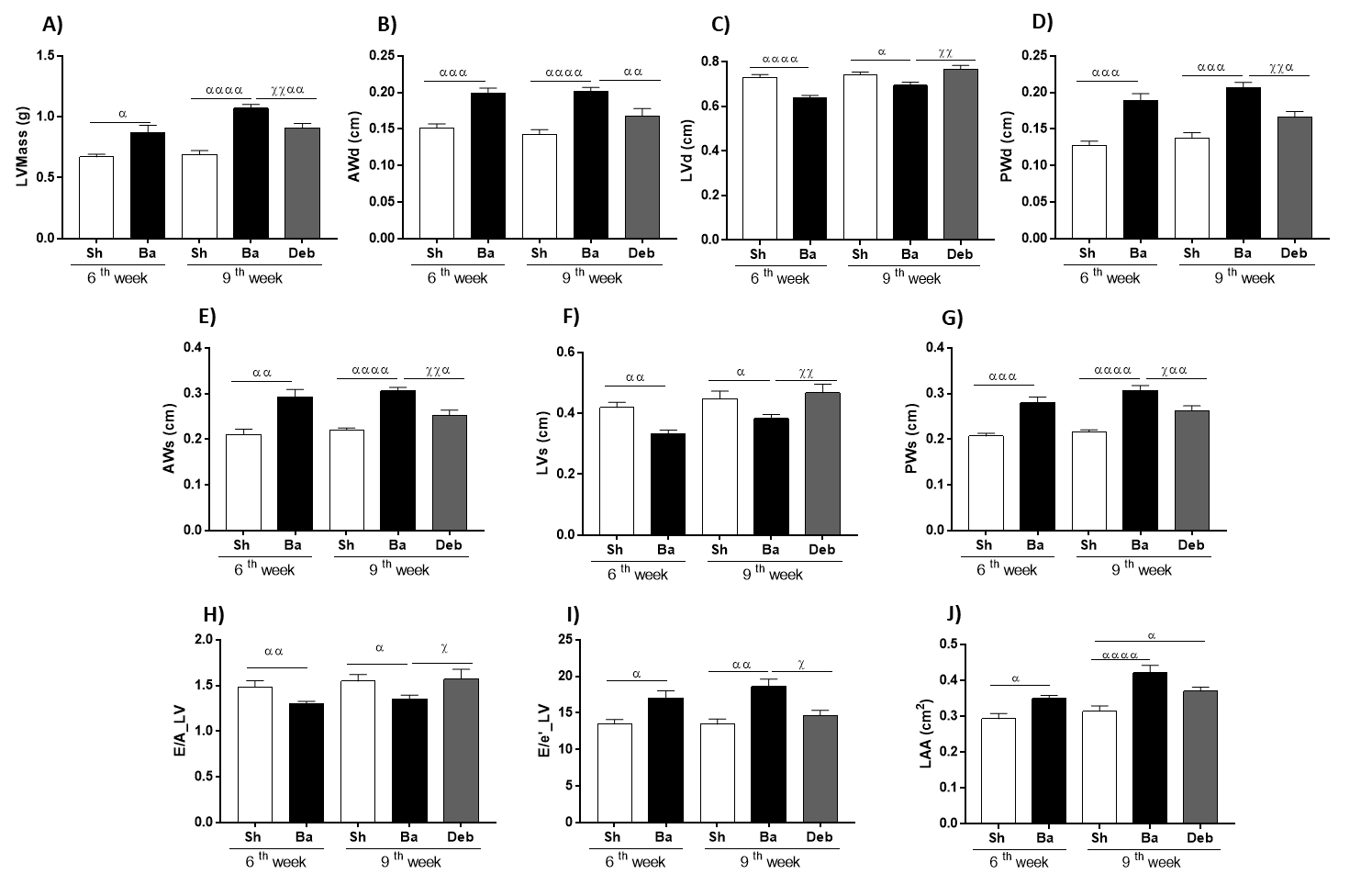** |
| --- |
| **Figure S1:** A) left ventricle mass, LVMass; B) anterior wall in diastole, AWd; C) left ventricle cavity in diastole, LVd; D) posterior wall in diastole, PWd; E) anterior wall in systole, AWs; F) left ventricle cavity in systole, LVs; G) posterior wall in systole, PWs; H) Ratio between peak of early to late mitral flow velocity of left ventricle, E/A; I) ratio between E and E’ waves, E/E’; J) left atrium area. LAA. Values are mean ± SEM, T-TEST for Sh 6^th^ vs Ba 6^th^ week, ANOVA for Sh 9^th^ vs Ba 9^th^ week. Ba/Deb vs Sh: α, p<0.05; αα, p< 0.01; ααα, p<0.001; Deb vs Ba: χ, p<0.05; χχ, p<0.01. |

**Supplements 1:**

**Supplements 2:**

| 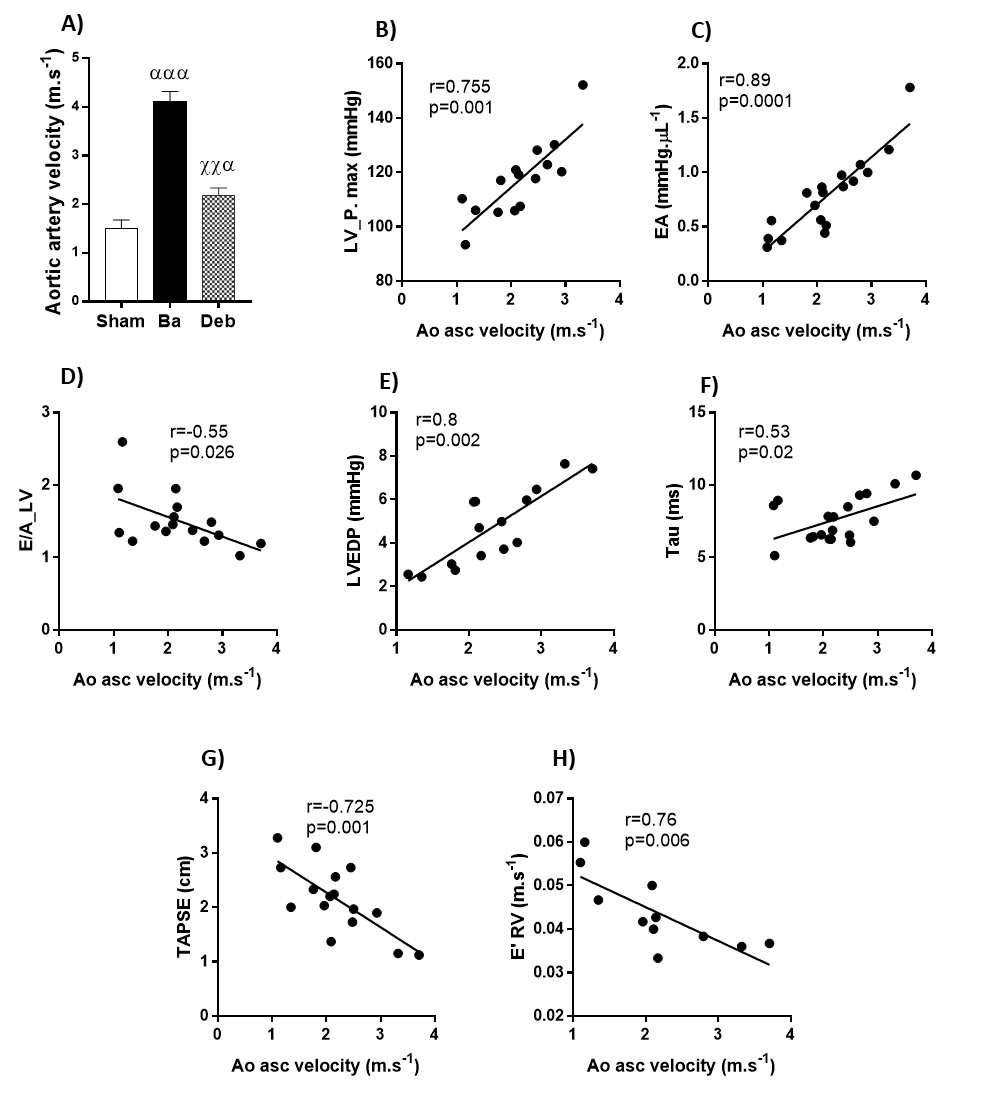 |
| --- |
| \| **Figure S2:** A) Differences in ascending aorta velocity among experimental groups; B-H) Correlations in debanding group between velocity of ascending aorta and: B) Left ventricle maximal pressure; C) Arterial elastance (EA); D) Ratio between peak of early to late mitral flow velocity of left ventricle (E/A); E) left ventricle end-diastolic pressure; F) Left ventricle time constant of relaxation; G) tricuspid annular plane systolic excursion (TAPSE) and H) Wave velocity of tissue Doppler at the lateral mitral annulus (E´). \| \| --- \| |

**Supplements 3:**

| **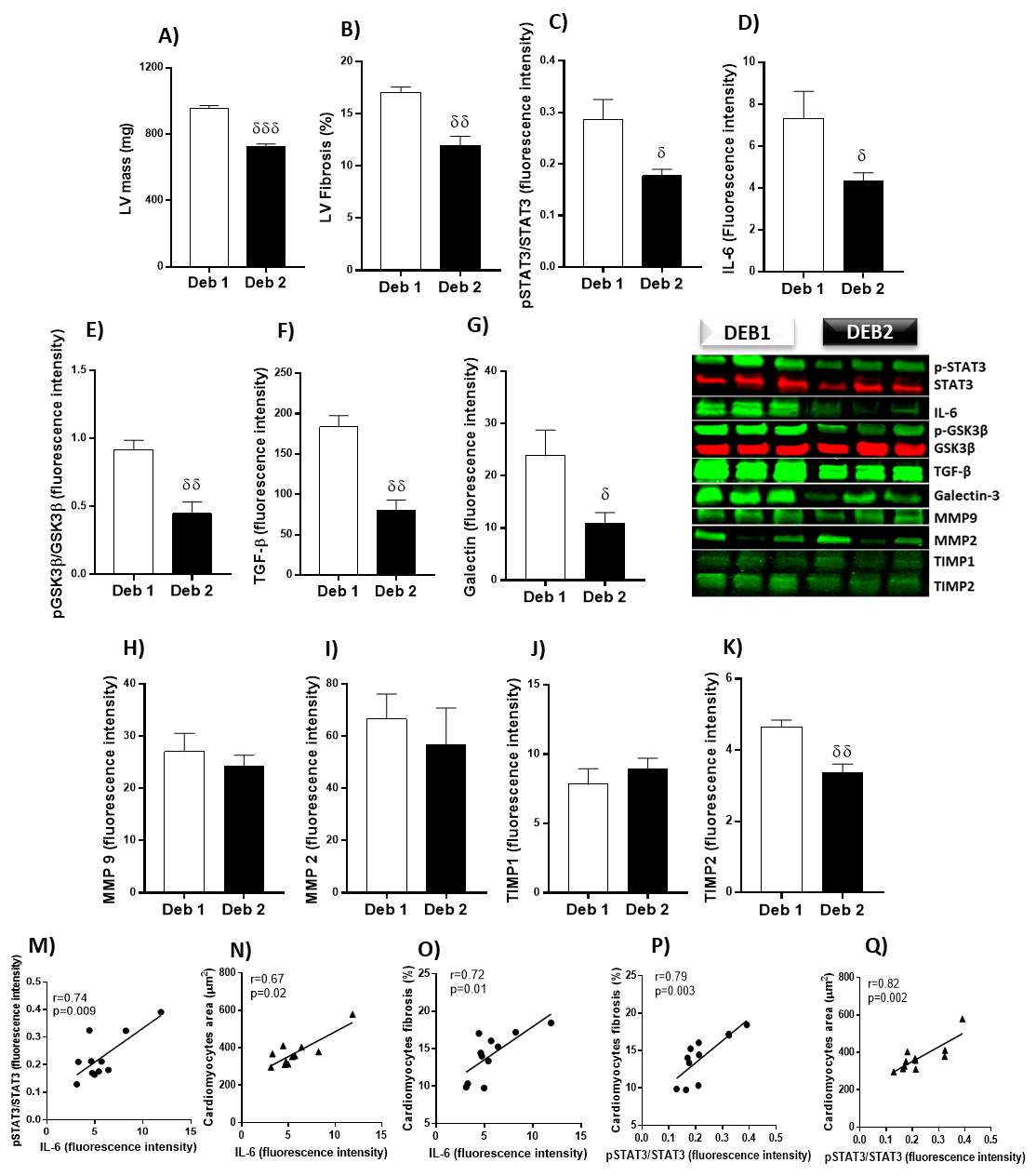** |
| --- |
| **Figure S3- Debanding sub-categorization and quantification of inflammatory and hypertrophic markers**. A) left ventricular mass; B) left ventricle fibrosis; C) ratio of phospho to total Signal transducer and activator of transcription 3; D) Interleucin-6; E) ratio of phosphorylated to total glycogen synthase kinase 3 beta (GSK3β); F) Transforming growth factor beta ; G) Galectin; H) matrix metalloproteinase 9; I) matrix metalloproteinase 2; J) tissue inhibitor of matrix metalloproteinases 1; K) tissue inhibitor of matrix metalloproteinases 2; M) correlations between IL-6 and pSTAT3/STAT3 intensity fluorescence determined by western blot; N-O) correlations between IL-6 intensity fluorescence (determined by western blot) and cardiomyocyte area and myocardial fibrosis; P-Q) correlations between pSTAT3/STAT3 intensity fluorescence (determined by western blot) and cardiomyocyte area and myocardial fibrosis. Values are mean ± SEM. δ, p<0.05; δδ, p< 0.01; Deb1 vs Deb2 |

**Supplements 4:**

| 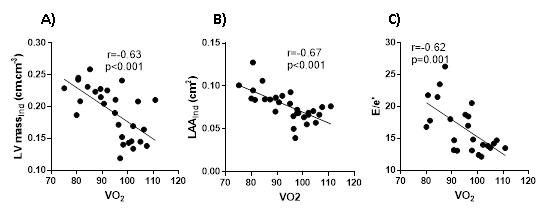 |
| --- |
| **Figure S4:** Correlations between maximum rate of oxygen consumption (VO_2_max) and: A) Indexed left ventricular mass (LVmass_ind_); B) Indexed left atrium area (LAA_ind_); C) Ratio between peak of pulse Doppler wave of early mitral flow velocity (E) and wave velocity of tissue Doppler at the lateral mitral annulus (E´) (E/e’). n=8 for each group. |

**Supplements 5:**


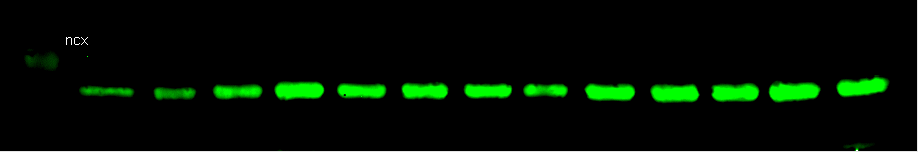

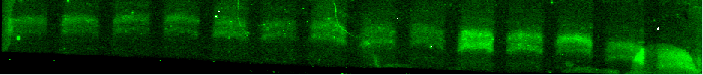


pSTAT3


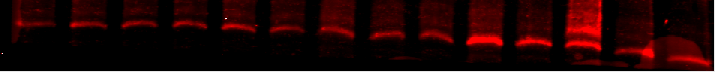


STAT3


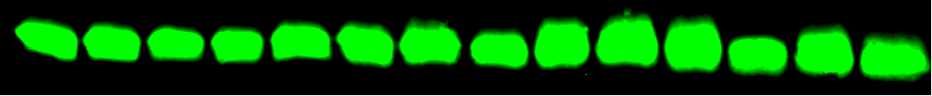


PLB


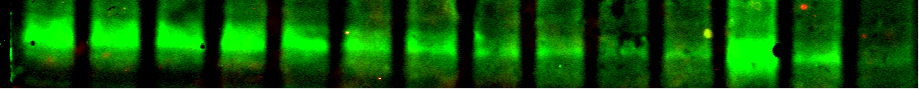


pCAMKii


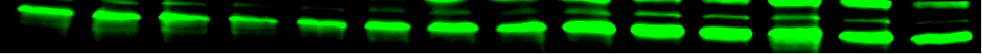


CAMKii


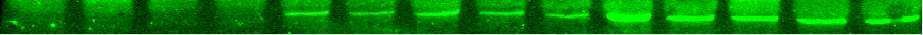


IL-6

NCX


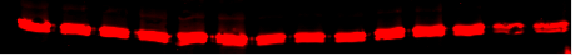


AKT


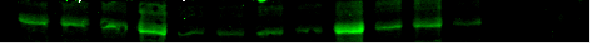


p-AKT


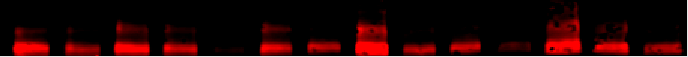


Col-I


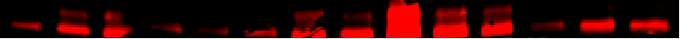


Col-III


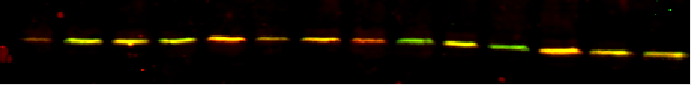


Total and

p-mTOR


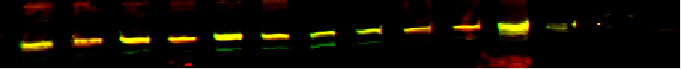

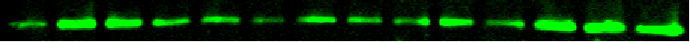


Serca


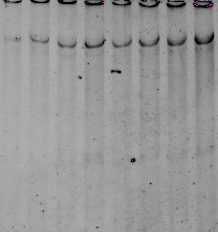

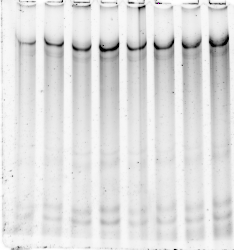


Sypro

ProQ


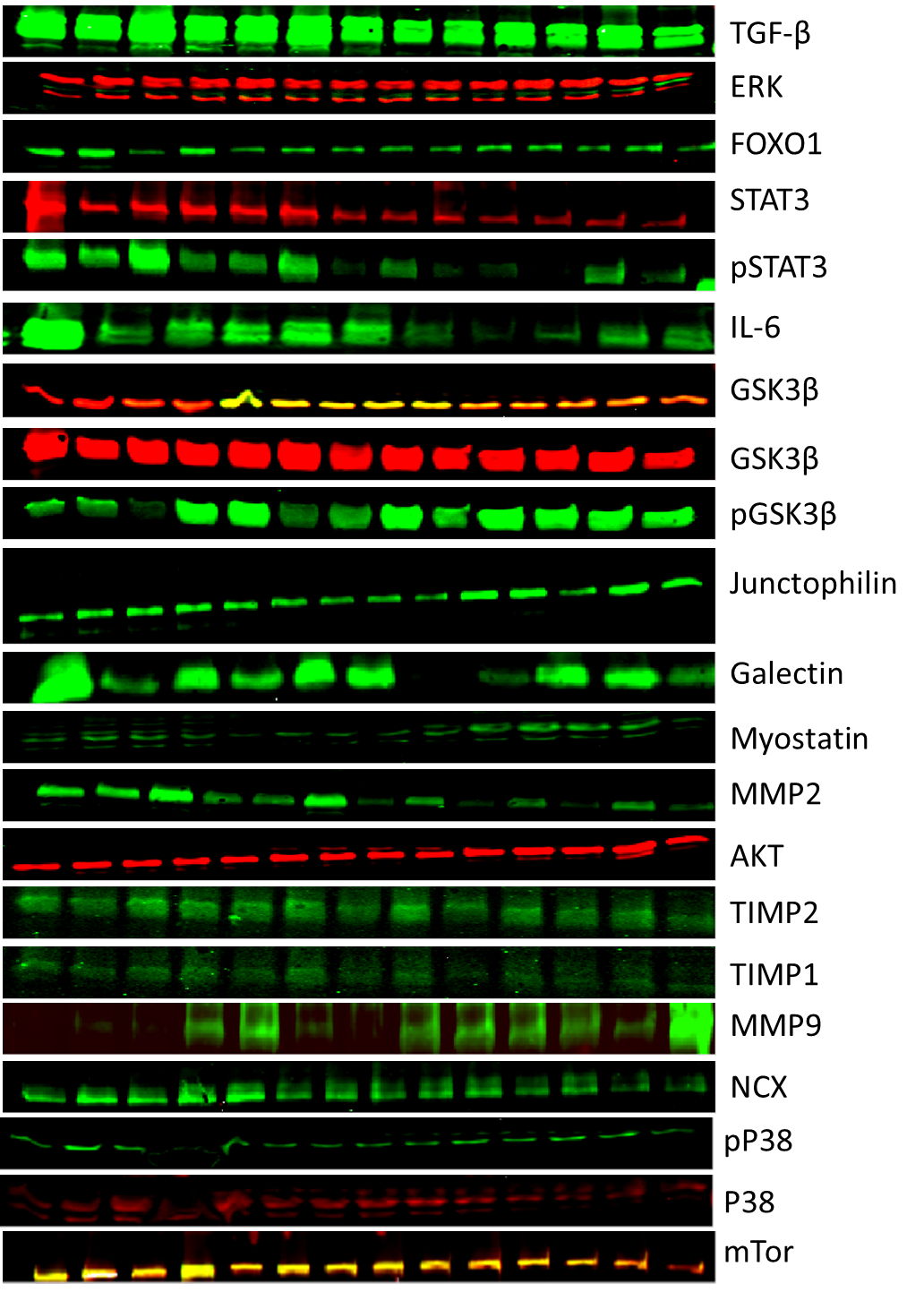

Supplement: Supplementary file 1 — Supplements SREP-18-36653A [file 41598_2019_39581_MOESM1_ESM.docx]
